# Impact of Clinical Intervention and Improper Storage on Amoxicillin-Clavulanate Effectiveness: A Case of Foot Infection in a Hot Climate

**DOI:** 10.7759/cureus.95743

**Published:** 2025-10-30

**Authors:** Manar A Alhujaili

**Affiliations:** 1 Pharmacology, Ministry of Health (MOH), Madinah, SAU

**Keywords:** amoxicillin-clavulanate, antibiotic stability, medication storage, non-adherence, pharmacist intervention

## Abstract

Amoxicillin-clavulanate is often used for skin and soft tissue infections because it works against a wide range of bacteria​. But if you store it improperly or skip doses, it may not work as well. This phenomenon is especially true in hot climates, where high temperatures speed up the breakdown of the drug. A young man visited the emergency pharmacy with a new prescription for metronidazole 500 mg three times a day to treat a foot infection that was getting worse. The pharmacist learned during counseling that another doctor had just prescribed amoxicillin-clavulanate 625 mg twice a day to the patient. The patient said that he left the antibiotic in his car in very hot weather and threw it away because he thought it had gone bad. The new prescriber did not receive this information, which led to incomplete treatment and the infection getting worse. This case shows how the environment and how a patient acts can change how well an antimicrobial works. Antibiotics can break down when they are exposed to heat, which makes them less stable and effective. Failing to follow the instructions can cause subtherapeutic concentrations and treatment failure. The pharmacist's help was crucial in finding these factors and suggesting ways to correct them. In hot climates, it is very important for antibiotics to stay stable and for patients to follow their treatment plan to achieve the best results. Interventions led by doctors and pharmacists are crucial for stopping unnecessary therapeutic failures and encouraging the right use of antibiotics.

## Introduction

Antimicrobial therapy is still essential for treating bacterial infections, but how it successfully works in practice depends on a number of factors, including the stability of the drugs, how well the patient responds to the therapies, and how effectively the healthcare professional is able to assist. Amoxicillin-clavulanate is a combination of a β-lactam antibiotic and a β-lactamase inhibitor. It is commonly used to treat skin and soft tissue infections because it is safe and works against a wide range of bacteria [1]. But the efficacy of this antibiotic can be greatly affected by how it is stored, especially in hot climates where high temperatures speed up the breakdown of the drug and lower its potency [2]. Maintaining antibiotic stability in areas with limited refrigeration can lead to ineffective dosing and treatment failure [3].

To stay stable, temperature-sensitive drugs like amoxicillin-clavulanate need to be kept below 25°C [4]. Too much heat can break down the β-lactam ring, which makes it less effective against bacteria [5]. In tropical or desert areas, the temperature outside often goes above this level, which means that things will probably break down unless certain storage steps are taken. Improper storage and insufficient patient counseling about medications are both factors contributing to therapeutic failure and the growing problem of antimicrobial resistance [6].

Unintentional non-adherence, such as skipping doses, taking them at the wrong time, or stopping treatment too soon, makes therapeutic outcomes even worse, in addition to physical stability. People often do not follow their treatment plans because they do not understand the instructions, they have side effects, or they think they have gotten better before finishing therapy [7]. These actions lower the effectiveness of drug exposure, which lets bacteria stay alive and raises the risk of recurrence or resistance [8]. Pharmacists and clinicians play a key role in reducing these risks by counseling patients, monitoring adherence, and taking prompt action when needed [9].

In hot and humid climates, foot infections are prone to accelerated bacterial proliferation, which can significantly impede the healing process. Improper drug storage and unintentional non-adherence can cause clinical failure, even when pathogens are initially susceptible [10].

This case report demonstrates that pharmacist-led intervention and patient education were essential in recognizing and addressing treatment failure related to amoxicillin-clavulanate degradation and non-adherence. The results highlight the necessity for awareness concerning antibiotic storage stability and adherence in elevated temperatures to enhance antimicrobial efficacy and avert resistance development.

## Case presentation

A healthy young man with an open wound on his left foot and acute pain was referred to the emergency pharmacy with a new prescription for metronidazole 500 mg orally three times daily and tetanus toxoid. The pharmacist identified a mismatch between the diagnosis and the prescribed treatment. After contacting the emergency physician to review the patient’s recent medication history, it was found that another emergency physician from the same hospital had prescribed amoxicillin-clavulanate 625 mg orally twice daily two days earlier. Upon further questioning by the first physician, the patient admitted that he had left the antibiotic in his car under extremely hot conditions exceeding 40°C. Believing the medicine had spoiled, he discarded it, but this information had not been communicated to the new prescribing physician. As a result, the infection worsened, leading to a return visit to the clinic, where only metronidazole was prescribed, unaware of the incomplete earlier therapy.

The pharmacist immediately recognized the risk of unintentional non-adherence and antibiotic degradation. After verifying the details with the patient and reviewing the clinical notes, the pharmacist contacted the physician to clarify the history and advised him to add amoxicillin-clavulanate again.

The treatment was adjusted to reinstate amoxicillin-clavulanate alongside metronidazole, with proper instructions on dosing, timing, and cool, dry storage. The pharmacist provided detailed counseling on the importance of completing the full antibiotic course. The pharmacist also stressed the importance of avoiding heat exposure and moisture during storage. The pharmacist also emphasized the importance of recognizing early signs of worsening infection. At follow-up, the wound had healed without further complications.

The pharmacist’s full clinical assessment and interventions are displayed in Table 1.

**Table 1:** Pharmacist’s clinical assessment and interventions

Category	Assessment	Action taken	Rationale/goal	Outcome
Prescription review	Monotherapy with metronidazole for foot infection	Contacted prescriber	Ensure appropriate antibacterial spectrum	Optimized therapy
Medication history	Previous antibiotic exposure	Verified with patient and medical record	Identify incomplete therapy	Clarified history
Patient counseling	Improper drug storage (heat exposure)	Educated on correct storage and adherence	Prevent recurrence, preserve efficacy	Patient understood and complied
Communication	Interprofessional collaboration	Notified physician, recommended combination therapy	Promote antimicrobial stewardship	Coordinated care
Follow-up	Healing evaluation	Reviewed progress	Confirm successful outcome	Infection resolved

## Discussion

Amoxicillin-clavulanate is one of the most commonly prescribed antibiotics for infections of the skin and soft tissue because it works against both Gram-positive and Gram-negative bacteria [1]. To keep antibiotics stable, the World Health Organization (WHO) says they should be stored below 25°C and kept away from moisture and sunlight [2]. Studies have demonstrated that prolonged exposure to temperatures exceeding 25°C accelerates the hydrolysis of both active components, resulting in substantial potency loss [3]. When these conditions are not satisfied, subtherapeutic concentrations may occur, possibly resulting in treatment failure and the development of resistant bacterial strains [4]. The clavulanate part, in particular, breaks down quickly when the temperature rises, which makes β-lactamase inhibition less effective and, as a result, makes the antibacterial activity less effective [5]. They characterize adherence as a dynamic process shaped by patient knowledge, health literacy, and the perceived significance of medication [7]. Taking medicine as prescribed would address a major global health problem, benefiting almost half of the people with chronic or infectious diseases [8]. Pharmacists are increasingly vital in ensuring patients take their medications correctly and that their treatments do not fail, and they do their job by teaching patients, following up with them, and doing clinical interventions [9]. In tropical climates like those in many parts of Africa and the Middle East, where the average daily temperature can reach 35°C or higher, it can be extremely difficult to keep things stored properly without refrigeration [10]. Providing a different way to discuss drug adherence by dividing it up into the following stages, beginning, continuing, and ending [11], people may fail to follow their treatment plan because they forget to take their medicine, misunderstand the instructions, or stop taking it too soon when their symptoms improve [12]. Research has demonstrated that pharmacist-led counseling enhances adherence rates and therapeutic success in outpatient settings by targeting these behavioral factors [13]. It has been shown that interventions like reinforcing dosing schedules, explaining storage needs, and counseling on the dangers of changing therapy on your own can improve outcomes [14]. Most of them overlook the period following drug administration, during which degradation and misuse can reduce the effectiveness of treatment [15]. Teaching patients how to handle their medications correctly is important for keeping antibiotics effective and stopping resistance from developing [16]. Some studies indicate that adding stabilizing agents to antibiotics or making coatings that can withstand high temperatures may help them last longer in tropical climates [17]. Moreover, heightened regulatory scrutiny regarding labeling mandates and patient directives can mitigate the hazards linked to inadequate storage [18]. Foot infections, particularly those linked to diabetes or trauma, frequently necessitate extended antibiotic treatment. In hot climates, where bacteria grow quickly and the environment is often contaminated, not getting the right treatment can have serious effects, from chronic infection to amputation [19].

This case underscores the vital interaction between drug stability, patient compliance, and prompt clinical intervention in achieving effective antimicrobial therapy. It also highlights the important key learning points and recommendations (Table 2). In this instance, the pharmacist's evaluation revealed both inadequate drug storage and insufficient compliance, facilitating immediate rectification of these concerns. Therefore, making sure that drugs stay stable, people stick to their treatment plan, and pharmacists become involved early on is crucial for getting favorable results from treatment.

**Table 2:** Key learning points and recommendations

Area	Learning points	Recommendations
Antibiotic stewardship	Importance of verifying recent antibiotic use	Encourage prescribers to review patient’s full medication history
Pharmacist role	Pharmacists can identify prescription-diagnosis inconsistencies	Empower pharmacists to intervene early
Medication storage	Heat exposure degrades antibiotics	Educate patients on proper storage (cool, dry, away from sunlight)
Communication	Interdisciplinary communication prevents therapy gaps	Maintain open lines between pharmacists and physicians
Patient adherence	Non-adherence due to misunderstanding	Reinforce patient education at dispensing time

## Conclusions

This case highlights how clinical intervention, patient education, and awareness of environmental factors can work together to correct treatment failure caused by improper medication use and storage. It emphasizes that effective antimicrobial therapy requires more than an accurate prescription; patients must also understand how to take and store their medication correctly. Pharmacists play a key role in addressing these issues through counseling and follow-up. In hot climates where improper storage is common, such interventions are essential to maintain antibiotic effectiveness and help prevent antimicrobial resistance.
